# Emergency Department Utilization by Pediatric Patients With Sickle Cell Disease in Basrah, Iraq

**DOI:** 10.7759/cureus.58277

**Published:** 2024-04-14

**Authors:** Ahmed S Marroof, Meaad K Hassan

**Affiliations:** 1 Department of Pediatrics, Al-Zubair general Hospital, Basrah Health Directorate, Basrah, IRQ; 2 Department of Pediatrics, College of Medicine, University of Basrah, Basrah, IRQ

**Keywords:** acute painful episodes, readmission, emergency department, children and adolescents, sickle cell disease

## Abstract

Background

Patients with sickle cell disease (SCD) often present in the Emergency Department (ED) with acute and debilitating pain and other SCD-related complications.

Objectives

The objective is to analyze the causes of ED visits of pediatric patients with SCD, assess the burden of ED admission due to SCD in relation to other pediatric diseases, the treatment given, and the outcomes.

Methods

A prospective analytical study was conducted on children and adolescents with SCD, 1-14 years old who had been admitted to the ED at Basrah Maternity and Children Hospital over a six-month period. Patient's sociodemographic and clinical data, drug history, length of ED stay, complications, outcome, and readmissions were recorded.

Results

A total of 422 patients with SCD were admitted to ED during the study period representing 4.10% of the total admitted cases; 276(65.40%) of them were recruited in this study, and their mean age was 7.84 ±3.47 years.

The main cause for ED admission was pain (73.91%), followed by infection (10.14%) and hemolytic crisis (6.15%). The mean duration of stay at ED was 6.11±1.87 hours. All admitted SCD patients had received analgesia; non-steroidal anti-inflammatory drugs (NSAIDs) were the commonest (80.4%), followed by acetaminophen (39.5%), and opioid narcotic (18.5%).

Readmission within 30-days was reported in 82(29.71%) patients and was associated with the number of ED visits/last year (B=0.151, P=0.023), length of stay at ED (B=0.140, P=0.034) and severe disease (B=0.253, P<0.001).

Conclusions

Acute painful episodes were the main cause of ED admission. Although most patients with pain did receive NSAIDs, only a small percentage of them did receive opioids. About one-third of patients have been readmitted within 30 days, and readmission was associated with the number of ED visits/last year, disease severity, and length of ED stay. These findings can help in establishing local guidelines for managing such patients in the ED especially pain management.

## Introduction

Sickle cell disease (SCD), one of the most common inherited disorders worldwide, is characterized by recurrent attacks of acute illness that affect different body organs leading to progressive organ damage and it represents a major public health problem as it is associated with high morbidity and mortality [1]. SCD is considered the most common inherited human blood disorder, affecting approximately 4.4 million individuals globally [2]. In the Basrah governorate in southern Iraq, 6.48% of the population are carriers of the sickle hemoglobin (HbS) gene, resulting in a gene frequency of 0.0324 [3].

Sickle cell anemia is distinguished by two main elements; namely vaso-occlusion and an accelerated rate of intravascular hemolysis, causing various pathological events, including inflammation, coagulopathy, platelet activation, vascular-endothelial dysfunction, and oxidative damage. This will lead to progressive vasculopathy and organ damage and will result in recurrent attacks of acute illness like acute painful episodes, acute chest syndrome (ACS), and stroke [4]. Furthermore, there is an increased risk of infections, acute splenic sequestration crises (ASSC), and chronic organ failure [4,5].

The improvements in the management of SCD have resulted in improved survival and decreased morbidity in these patients especially in developed countries; however, much less progress has been made in the developing world, including the Mediterranean region, where the disease is common, and is still considered an important cause of childhood mortality [5].

The main presentation of SCD is recurrent attacks of acute and severe pain associated with vaso-occlusive crisis (VOC) that often requires emergency department (ED) consultation and admission. Patients with SCD may also present in the ED with other SCD-related complications like exacerbation of anemia, ACS, ASSC, infections, priapism, stroke, and other end-organ damage [6,7]. These complications are considered challenges for crowded EDs as these patients require rapid evaluation and management for specific life-threatening conditions [8].

Taking into consideration being both an acute and chronic disease, SCD is associated with significant health care utilization. Factors that were found as predictors of multiple ED visits by SCD patients included age ≥ 21 years, and insurance status (more in uninsured patients) [9]. In Brazil, as a middle-income country, acute painful episodes were responsible for 64% of all visits to the ED with a median cost of $127.11/visit, followed by infections, exacerbation of anemia, and organ damage. Exacerbation of anemia was the most expensive cause of ED visits ($321.87/visit), followed by chronic organ damage ($236.40/visit) [10].

Optimum and immediate management of acute SCD-related complications is considered the mainstay of clinical care [6]. Improvement in the ED care of patients with SCD is of paramount importance in managing and improving the quality of life of these patients. Therefore, we have conducted this study to analyze the main causes of emergency department visits of pediatric patients with SCD in Basrah, study the socio-demographic and clinical characteristics of SCD-admitted patients, assess the burden of ED admission due to SCD in relation to other pediatric diseases, the treatment given and outcome of admitted SCD patients.

## Materials and methods

A prospective analytical study was carried out on children and adolescents with SCD, who had been admitted for different reasons to the ED at Basrah Maternity and Children Hospital from the first of August 2018 through January 2019. The total number of pediatric patients who have been admitted to the ED during the study period was also evaluated. A total of 422 patients with SCD were admitted to ED during the study period; 276 (65.40%) of them were recruited in this study. The rest were excluded either because they were not seen and/or followed by the same researchers or the patients had not been registered at the CHBD.

Patient's data including age, gender, residence, date of admission, parental educational level, number of other affected siblings, and main reasons for consulting the ED were recorded. The past medical history included age at diagnosis, frequency of follow-up outpatient visits, number of ED admissions/last year, and the number of blood transfusions/last year. Data also included the genotype of SCD, number of outpatient visits to the CHBD and whether these visits are regular or irregular, medications taken (hydroxyurea or penicillin), length of ED stay (hours), complications, outcome, and readmissions if present. Two timeframes are used for identifying readmissions: seven days and 30 days after discharge of an initial stay.

Patients were examined thoroughly, and the severity of pain was assessed by the Faces, Legs, Activity, Cry, and Consolability (FLACC) used for infants and young children, and the Visual Analogue Scale (VAS 0-10) for older children. 

Definition of variables

The family income was classified according to the average monthly per capita (which measures the income earned by each individual) in Iraqi Dinars (ID) into low (<100.000 ID/month), medium (100.000-250.000 ID/month), and high (>250.000 ID/month) [11].

Severe disease was defined as frequent VOC requiring hospitalization ≥3 /year, blood transfusion ≥3/year, frequent hospitalization ≥3/year, an episode of stroke, ACS, or avascular necrosis of bone (AVN) [12]. 

Patients were considered to have hemolytic crises if there was an acute reduction in Hb level, associated with increased reticulocyte count higher than the baseline count. It can occur in the following situations: delayed hemolytic transfusion reaction, autoimmune hemolysis, and infections [13].

Irregular Visits

The CHBD follows a schedule of visits every two to three months in the first two years of life. After the age of two, the frequency of visits depends on the patient's family needs and access to a medical consultation, but it should be at least, every six months Patients who had provided OPD appointments for follow-up but didn’t show up are considered to have irregular visits [14].

Statistical analysis

Data were analyzed using the Statistical Package for Social Science (SPSS) program version 24 software (IBM Corp., Armonk, NY, USA). Data were expressed by number (N) and percentages (%) or means ± standard deviation (SD). The chi-squared test and Fisher’s exact test were used to assess categorical variables (presented as numbers and percentages). The independent t-test was used for quantitative comparison between two means of different samples. Analysis of the independent risk factors associated with readmission to ED was evaluated using logistic regression analysis. For all tests, the level of significance was considered when the P-value was <0.05. 

## Results

The total number of patients who had been admitted to the ED at Basrah Maternity and Children Hospital during the study period was 10,291 patients; out of this number (422) were children and adolescents with SCD, representing 4.10% of the total ED admitted cases. Out of these 422 patients with SCD; 276 (65.40%) of them were recruited in this study; 172 (62.32%) males and 104 (37.68%) females. The age of the admitted patients ranged from 1 to 14 years with a mean age of 7.84 ±3.47 years and a median age of 8 years. Most of ED admitted SCD patients had severe disease (61.23%), were with S/β thalassemia (59.78%), aged 6-10 years (39.49%), residing in the center of Basrah governorate (62.68%) and around two-thirds of admitted patients were males (male: female ratio was 1.65:1). Furthermore, around two-thirds of parents of ED admitted SCD patients were of low educational level, and only 12% of them were with high monthly income. The majority of admitted patients (91.67%) live with both parents, and almost 40% have another sibling with SCD (Table 1).

**Table 1:** Selected socio-demographic characteristics of SCD patients admitted to Emergency Department Data were expressed as N. (%). Abbreviations: SCA: sickle cell anemia; S/β-Thalassemia: Sickle/β-Thalassemia

Variable		SCA Total 111	S/βThalassemia Total 165	Total 276
		N. (%)	N. (%)	N. (%)
Age (year)	< 2	7 (6.31)	11 (6.67)	18 (6.52)
	2- 5	23 (20.72)	40 (24.24)	63 (22.83)
	> 5-10	44 (39.64)	65 (39.39)	109 (39.49)
	>10	37 (33.33)	49 (29.70)	86 (31.16)
Gender	Male	73 (65.77)	99 (60.00)	172 (62.32)
	Female	38 (34.23)	66 (40.00)	104 (37.68)
Residence	Periphery	42 (37.84)	61 (36.97)	103 (37.32)
	Center	69 (62.16)	104 (63.03)	173 (62.68)
Education level of father	Illiterate	22 (19.82)	47 (28.48)	69 (25)
	Primary	51 (45.95)	64 (38.79)	115 (41.67)
	Secondary	28 (25.23)	35 (21.21)	63 (22.83)
	Higher	10 (9)	19 (11.52)	29 (10.50 )
Education level of mother	Illiterate	26 (23.42)	55 (33.33)	81 (29.34)
	Primary	55 (49.51)	61 (36.96)	116 (42.02)
	Secondary	23 (20.72)	39 (23.63)	62 (22.46 )
	Higher	7 (6.31)	10 (6.06)	17 (6.15 )
Parental status	Live together	105 (94.59)	148 (89.70)	253 (91.67)
	Divorced	3 (2.71)	8 (4.85)	11 (3.98)
	Death of one of parents	3 (2.70)	9 (5.45)	12 (4.35)
Housing condition	Owned	62 (55.86)	100 (60.60)	162 (58.69)
	Rented	49 (44.14)	65 (39.40)	114 (41.31)
Family income	<100,000	52 (46.85)	80 (48.48)	132 (47.83)
	100,000-200,000	45 (40.54)	66 (39.29)	111 (40.22)
	>200,000	14 (12.61)	19 (11.53)	33 (11.95)
No. of affected family members	None	71 (63.96)	94 (56.97)	165 (59.78)
	1	31 (27.93)	46 (27.88)	77 (27.90)
	2	6 (5.41)	20 (12.12)	26 (9.42)
	3	2 (1.80)	4 (2.42)	6 (2.17)
	4	1 (0.90)	1 (0.61)	2 (0.72)
Caregivers	Father	48 (43.24)	54 (32.73)	102 (36.96)
	Mother	41 (36.94)	85 (51.51)	126 (45.65)
	Others	22 (19.82)	26 (15.76)	48 ( 17.39)

The main cause for ED admission among SCD was pain (73.91%), followed by infection (10.14%) and hemolytic crisis (6.15%) (Table 2). In 195 (70.65%) patients, the pain was acute due to VOC, and in nine (3.26%) it was due to AVN. The study also showed that pain was responsible for recurrent ED admissions in 59 (21.38%) patients. Concerning infections, 12 (4.34%) patients had UTI, seven (2.53%) acute diarrhea, four (1.44%) hepatitis A, four (1.44%) tonsillitis and one patient had acute cholecystitis (0.36%), none of patients was proved to have sepsis. There is no significant difference between both types of SCD regarding the causes of admission, P>0.05 (Table 2).

**Table 2:** Diagnosis of SCD patients at admission to emergency department in relation to type of SCD † Chi square was used to assess P-value, *P-value was assessed by Fisher’s exact test.

Diagnosis	SCA Total 111; N (%)	S/ β thalassemia Total 165; N (%)	Total 276; N (%)	P-value
Acute painful crisis	79 (71.17)	125 (75.76)	204 (73.91)	0.395^†^
ACS	8 (7.21)	8 (4.85)	15 (5.43)	0.287^†^
ASSC	7 (6.31)	3 (1.82)	10 (3.63)	0.095*
Hyper hemolytic crisis	5 (4.51)	11 (6.67)	17 (6.16)	0.348^†^
Infection	10 (9.01)	18 (10.91)	28 (10.14)	0.608^†^
Stroke	2 (1.80)	0 (0)	2 (0.72)	0.161*

Although most of admitted patients did not develop complications 158(57.25%), a considerable percentage of cases were complicated by ACS (15.21%), infections (13. 04%), ASSC (9.78%), and AVN (3.26%). Four patients (1.44%) were found to have stroke while in the ED. Infections were reported in a significantly higher frequency among S/β-thalassemia patients 27 (16.36%) compared to SCA patients nine (8.11%), P=0.046.

The study also revealed that most of SCD patients (64.14%) had regular visits to the CHBD which are mostly ≥ 3 visits per year, and around quarter of them did receive frequent blood transfusions ≥ 3/year. It also revealed that the majority (67.03%) were not on HU therapy and that patients with S/β thalassemia did receive significantly more blood transfusions and HU compared to those with SCA, P<0.05 (Table 3).

**Table 3:** Long-term follow up and care of studied patients *Chi-square test was used to assess P-value.

Variable		SCA Total 111	S/β thalassemia Total 165	Total 276	P-value*
		N. (%)	N. (%)	N. (%)	
Follow up visit	Regular	64 (57.66)	113 (68.48)	177 (64.14)	0.066
	Irregular	47 (42.34)	52 (31.52)	99 (35.86)	
Frequency of CHBD visit/ last year	None	14 (12.61)	15 (9.10)	29 (10.50)	0.446
	1-2	26 (23.43)	33 (20)	59 (21.38)	
	≥ 3	71 (63.96)	117 (70.90)	188 (68.12)	
N. of blood transfusion/ last year	None	49 (44.15)	45(27.27)	94(34.05)	0.015
	1-2	37 (33.33)	74 (44.85)	111 (40.22)	
	≥ 3	25 (22.52)	46 (27.88)	71 (25.73)	
HU therapy		18 (16.22)	48 (29.09)	66 (23.92)	0.014
Prophylactic Penicillin		39 (35.13)	52 (31.52)	91 (32.97)	0.530

The mean duration of stay at ED was 6.11±1.87 hours with no significant difference between those with SCA and S/β thalassemia, P>0.05. However, a significantly higher percentage of patients with S/β thalassemia were discharged home well while higher percentage of those with SCA were discharged on family responsibility, P<0.05. A total of 179 (64.86%) were admitted to the hospital, none of these patients required ICU, and there was no death. Out of the 276 patients with SCD; 29.71% were readmitted within 30 days. Readmission was highest during the first week following discharge from the ED; 52 were readmitted within one week which represented 63.41% of all the readmitted patients. The most common causes for readmission were acute painful episodes 59 (21.38%), followed by ACS nine (3.26%) and infections nine (3.26%) (Table 4).

**Table 4:** Length of stay at emergency department, outcome, and readmission of studied SCD patients *P-value was assessed by Fisher’s exact, **P-value assessed by independent T-test, †P-value assessed by Chi square.

Variable		SCA N. 111	S/β thalassemia N. 165	Total 276	P-0value
		N. (%)	N. (%)	N. (%)	
ED stay (hours)	< 2	2 (1.80)	11 (6.67)	13 (4.71)	0.052*
	2-6	34 (30.63)	60 (36.36)	94 (34.05)	
	6-8	75 (67.57)	94 (56.97)	169 (61.24)	
	Mean ± SD	6.40±1.58	5.92±2.03	6.11±1.87	0.036**
Outcome	Inpatient admission	76 (68.47)	103 (62.42)	179 (64.86)	0.011†
	Discharged well	19 (17.12)	54 (32.73)	73 (26.44)	
	Discharged on family responsibility	16 (14.41)	8 (4.85)	24 (8.70)	
Readmission					
No readmission		74 (66.67)	120 (72.73)	194 (70.29)	0.279†
Readmission	Within 1 month	37 (33.33)	45 (27.27)	82 (29.71)	0.447†
	Within 1 week	20 (16.22)	32 (19.39)	52 (18.84)	
Causes of readmission	Acute painful crisis	30 (81.09)	29 (64.44)	59 (21.38)	0.914†
	ACS	7 (18.90)	2 (4.44)	9 (3.26)	0.091*
	ASSC	3 (8.11)	1 (2.22)	4 (1.44)	0.329*
	Infection	1 (2.70)	8 (17.77)	9 (3.26)	0.011*
	Stroke	1 (2.70)	0 (0.00)	1 (0.36)	0.972*

Using logistic regression analysis, independent risk factors associated with readmission were the number of ED visits/last year, length of stay in ED and severe disease, P<0.05 (Table 5).

**Table 5:** Independent risk factors associated with readmission

Variable	Beta coefficient	P-value
Age	-0.107	0.133
Gender	0.031	0.638
Causes of ED admission	0.016	0.804
Age at diagnosis	0.047	0.492
Father education	-0.051	0.574
Mother education	0.054	0.528
Follow up visit	0.056	0.396
Frequency of ED visit/year	0.151	0.023
Use of Hydroxyurea	-0.022	0.750
Family income	0.033-	0.664
Length of stay in ED	0.140	0.034
Type of SCD	-0.047	0.476
Use of multiple utilization	-0.013	0.847
Frequency of outpatients visit/year	-0.039	0.562
Disease severity	0.253	<0.001

The majority of patients with SCD who were admitted to the ED had received intravenous fluids (69.2%), all had received analgesia and (31.5%) required oxygen. Non-steroidal anti-inflammatory drugs were the commonest (80.4%), followed by acetaminophen (39.5%), and opioid narcotic (18.5%). All patients given opioid narcotic had received prior NSAIDs. Most patients have consulted Basrah Maternity and Children Hospital only, although patients with S/β thalassemia and those residing at the peripheries had significantly more multiple utilization compared to those with SCA and those living at the center of Basrah, P<0.05 (Table 6).

**Table 6:** Healthcare facilities utilization of emergency department admitted patients Chi-square test was used to assess P-value

Variable		Utilization		Total (N.276)	P-value
		Multiple N.58	Single N.218		
		N. (%)	N. (%)	N. (%)	
Residence	Central	11 (18.97)	107 (49.08)	118 (42.75)	<0.001
	Periphery	47 (81.03)	111 (50.92)	158 (57.25)	
Type of SCD	SCA	15 (13.51)	96 (86.49)	111 (40.22)	0.012
	S/β thalassemia	43 (26.06)	122 (73.94)	165 (59.78)	

## Discussion

SCD is one of the common health problems in this part of Iraq. ED plays a pivotal role in the provision of immediate care for such patients. Besides managing acute pain, the commonest presentation of SCD, doctors working in the ED should be aware of other SCD presentations and disease-related complications that carry a significant risk of morbidity and mortality [6]. In this study patients with SCD constituted 4.10% of the total number of patients who had been admitted during the study period to the EDs at Basrah Maternity and Children, with a mean duration of stay at ED of 6.11±1.87 hours. This percentage of admitted cases to the ED out of all admitted cases is lower than that reported by Abhulimhen-Iyoha et al., in Nigeria (11.9%) [15]. In Brazil, Fernandesa et al. reported an increase in the total number of hospitalizations of SCD children < 14 years old relative to the total of hospitalizations of children of the same age group from 0.12% in 1999 to 0.37% in 2012 [16].

The analysis of sociodemographic risk factors for ED utilization of SCD patients in the current study revealed that most patients with S/β thalassemia, aged >5 years, residing in the center of Basrah governorate, and around two-thirds of admitted patients were males. The mean age of admitted patients and male preponderance is comparable to that reported by Brown et al. in Nigeria (7.3±4 years and 1.3:1, respectively) [17]. Other studies by de Medeiros Fernandes et al., in Brazil [18], and Attell et al., in the USA, also reported a male preponderance of ED-admitted SCD patients [19]. These gender-specific differences observed, partly already known in the adult, have been attributed to hormonal changes. However, in pediatric patients, other factors must be implicated but these factors have not been determined yet [20].

The current study found that around two-thirds of parents of ED-admitted SCD patients were of low educational level, and only 12% of them had with high monthly income. This finding is similar to that found by Fernandes et al. in Brazil [18]. Socio-economic factors are important determinants of the health of people in general and increased poverty adversely affects health. This is particularly relevant to patients with SCD which is prevalent mainly in poor countries [21]. 

The most common cause of ED visits among our SCD pediatric patients was acute painful crises among (73.91%) of patients. This result is similar to that reported by other researchers in different countries [10,19,22]. Initially, it was believed that the change in flow characteristics and erythrocyte aggregation alone caused vaso-occlusion and pain, however, the cause of vaso-occlusion is multifactorial. Endothelial activation with increased adhesion of erythrocytes and leukocytes followed by the formation of heterocellular aggregates, which result in occlusion and local hypoxia and also trigger a vicious cycle of increased HbS formation caused by hypoxia, presence of inflammatory mediators, free radicals, and reperfusion injury [6]. 

Acute painful episodes due to vaso-occlusion are the main cause for seeking ED medical care among patients with SCD; high doses of intravenous opioids are usually needed to treat such crises usually in the ED. Treatment of pain is recommended to be initiated within 60 minutes of arrival to the ED, reassessment within 30 minutes, and additional intravenous opioid administration if the pain is uncontrolled [23]. All pediatric SCD patients who had been admitted to the ED of Basrah Maternity and Children Hospital had received analgesia. NSAIDs (mainly Diclofenac sodium) were the most commonly used analgesic, followed by acetaminophen and opioid narcotics. Almahmoud et al., in Saudi Arabia, reported pain in 64.6% of the pediatric patients admitted to ED, and that morphine was given to 39.6% of patients with pain, followed by ibuprofen for 33% and acetaminophen given to 20% [22]. In the USA, narcotic drugs were given to 48% of pediatric SCD patients admitted to ED, followed by NSAIDs alone in 33% and the rest (19%) received both opioid narcotics and NSAIDs [24]. There is no established hospital policy to give NSAIDs as the first line before opioids, however, because of economic constraints and occasional shortages in opioid supply, NSAIDs are given initially to treat acute painful episodes. Unfortunately, morphine was not always accessible, and intranasal fentanyl is not available for managing pain at the ED of Basrah Maternity and Children's Hospital. 

Intravenous fluids were given to most of the patients admitted to the ED in this study. The treatment of VOC is complex, and it requires multiple interventions. Patients with SCD and acute painful episodes usually receive additional fluids (oral or intravenous), regardless of the hydration status of the patient, aiming to slow or stop the sickling process, decreasing blood viscosity and indirectly reducing RBC dehydration and intracellular concentration of HbS; however, the effectiveness of fluid therapy was not proven through randomized controlled trials [25].

Hydroxyurea, a myelosuppressive agent, became a standard of care to prevent both acute and chronic complications of SCD, improved quality of life, and prolonged survival. Hydroxyurea is an effective drug to reduce the frequency of painful episodes. It raises the level of HbF and hemoglobin level, decreases the rate of ACS episodes, hospitalizations, and blood transfusions as it ameliorates anemia and decreases hemolysis [26]. In the current study around 24% of patients were on HU therapy, this relatively low percentage can be attributed to the refusal of caregivers to start HU and occasional shortage in hydroxyurea supply. We think that clinicians should increase awareness and provide better education and counseling to the families about the role of HU in modifying SCD severity.

Early diagnosis of infections in SCD and timely institution of appropriate treatment are very important in reducing morbidity and mortality [17]. Infections were the second cause of ED visits among patients with SCD in our study. Patients with SCD are susceptible to infections for different reasons including splenic dysfunction, defects in opsonization of encapsulated organisms, impaired adaptive immunity, and immune deficiencies associated with malnutrition [27]. The frequency of infections among ED-admitted pediatric patients with SCD differs from one country to another. Brown et al., in Nigeria, reported infections in 72.4% of hospitalized children [17]. While Jain et al., in India, reported that infection was the main cause of admission in under 5 children with SCD [28]. Infections remain an important cause of morbidity and mortality in patients with SCD in low- and middle-income countries due to increased exposure to pathogens, increased co-morbidities such as malnutrition, lower vaccination rates, and less access to medical care, including antibiotics and blood. While in high-income countries; perinatal diagnosis of SCD, antimicrobial prophylaxis, vaccination, aggressive use of antibiotics for febrile episodes, and the availability of optimum intensive care resources have led to a significant reduction in deaths from infection [27].

No death was reported in the current study among the studied patients. Abhulimhen-Iyoha et al., in Nigeria, reported a mortality rate of 2.7% among pediatric SCD patients [15], and Brown et al., in Nigeria, also reported a mortality rate of 1.9% [17]. This may be related to disease severity and type of sickle haplotype prevalent in Africa.

A considerable percentage of our SCD patients were readmitted within one month following discharge from the ED, with around two-thirds of them being readmitted within one week. The frequency of return to the ED within one month in this study is comparable to that reported by Brousseau et al., in the USA; 22.3% among one to nine years old and 32.8% among patients 10 to 17 years old [29]. In Brazil, Fernandes et al. reported that hospital readmissions in 16.7% of children, with one-third of readmissions occurring within seven days after hospital discharge were more frequent in children with S/β thalassemia (P < 0.05) [16]. It has been suggested that follow-up of these patients in the outpatient shortly after discharge from the ED may prevent readmission of these patients [29]. The high readmission rate in our study can be attributed to uncontrolled pain, suboptimal pain management, short ED stay, severe SCD, and missing outpatient department visits. 

The most common causes for readmission were acute painful episodes, followed by ACS and infections. A significant positive association between the number of ED visits/last year, disease severity, and length of stay at ED with readmission was reported in the current study. Frei-Jones et al., in the USA, reported that the most common cause of admission and readmission in children with SCD was pain, 78% and 70%, respectively [30]. Education regarding the ambulatory use of oral analgesics is provided routinely to all patients and/or their caregivers in our center. Patients with recurrent acute painful episodes are also advised to start HU therapy. Frei-Jones et al. also found a significant association between disease severity and follow-up visits with readmission [30]. This can be attributed to that patients with severe disease have more disease-related complications which increase the number of possible admissions to ED.

Pediatric patients residing at the peripheries had utilized multiple healthcare facilities significantly more than those living at the center of Basrah. This can be explained by that patients residing in the center of Basrah consult the Center for Hereditary Blood Diseases directly when they develop SCD-related complications, while those living in the peripheries consult local healthcare facilities before coming to the Center for Hereditary Blood Diseases. 

Limitations of the study

There are many limitations of this study; as acute painful episodes are the main cause of ED admission, one limitation is that the time to initial pain assessment and treatment, reassessment of pain, and criteria to discharge patients from ED were not evaluated. Furthermore, because of the low number of patients on HU, its role in decreasing SCD-related complications mainly pain, and the need for ED visits were not evaluated also.

## Conclusions

This single-center study provided a review of the characters of pediatric SCD patients, and the services provided to them in the ED. Acute painful episodes were the main cause of ED admission; however, most of them did receive NSAIDs and only a small percentage of them did receive opioids. The study also evaluated readmission of pediatric patients with SCD within seven and 30 days, and readmission was associated with the number of ED visits/ last year, disease severity, and length of stay at ED. The findings of this study can help in improving services provided for such patients in the ED and in establishing local guidelines for managing SCD pediatric patients in the emergency setting, especially pain management.
